# Supplementary material for: Soil structure is an important omission in Earth System Models
Source: Nat Commun. 2020 Jan 27;11:522. doi: 10.1038/s41467-020-14411-z (PMC6985222; doi:10.1038/s41467-020-14411-z)
Supplement: Supplementary file 1 — Supplementary information [file 41467_2020_14411_MOESM1_ESM.pdf]

Supplementary information for

Soil structure – an important omission in Earth System Models

Simone Fatichi[1*], Dani Or[2,3], Robert Walko[4], Harry Vereecken[5], Michael H. Young[6], Teamrat Ghezzehei[7], Tomislav Hengl[8], Stefan Kollet[5], Nurit Agam[9], Roni Avissar[4]

[1]Institute of Environmental Engineering, ETH Zurich, Switzerland, simone.fatichi@ifu.baug.ethz.ch
[2]Department of Environmental Science, Institute of Biogeochemistry and Pollutant Dynamics, ETH Zurich, Zurich, Switzerland
[3]Desert Research Institute, Reno, Nevada, USA
[4]Rosenstiel School of Marine & Atm. Science, University of Miami, USA
[5]Agrosphere, Jülich Research Center, Germany
[6]Bureau of Economic Geology, The University of Texas at Austin, USA
[7]Life & Environmental Sciences, University of California, Merced, USA
[8]Envirometrix Ltd, the Netherlands
[9]Blaustein Institutes for Desert Research, Ben Gurion University of the Negev, Israel
*Corresponding Author

Supplementary Table 1. Site characteristics (latitude, longitude and biome type), simulated Gross Primary Production (GPP, [gC m^{-2} yr^{-1}]), and maximum root depth (R_{dp} [m]) and soil depth (Z_s [m]) used for the simulations.

Site	*Biome*	*Lat*	*Lon*	*GPP*	R_{dp}	Z_S
**Chamau (CH)**	C3 Grassland	47.21	8.41	2463	0.25	1.3
**Stubai (AT)**	C3 Grassland	47.12	11.32	1641	0.25	0.8
**UMBS (MI, USA)**	Deciduous Forest	45.56	-84.71	1252	2	3
**Manaus km34 (BR)**	Tropical Forest	-2.61	-60.21	3001	10	14
**Konza Prairie (KS, USA)**	C3/C4 Grassland	39.10	-96.60	1811	0.4	2.5
**Hyytiala (FI)**	Evergreen Forest Boreal	61.85	24.30	999	0.7	0.8
**Sevilleta grassland (NM, USA)**	C4 Grassland	34.36	-106.70	289	0.9	4
**Sevillata shrubland (NM, USA)**	Shrubs and C4 Grassland	34.33	-106.74	188	0.9	4
**ORNL FACE (TN, USA)**	Deciduous Forest	35.90	-84.33	1783	1	1.5
**Duke Forest (NC, USA)**	Evergreen Forest (Mostly)	35.96	-79.10	2114	0.5	3
**Harvard Forest (MA, USA)**	Deciduous Forest	42.54	-72.17	1513	1	2
**Morgan Monroe Forest (IN, USA)**	Deciduous Forest	39.32	-86.41	1557	1.8	2.2
**Short Grass Steppe (CO, USA)**	C3/C4 Grassland	40.81	-104.75	372	0.45	2.5
**Willow Creek (WI, USA)**	Deciduous Forest	40.81	-90.08	1157	1.2	1.4
**Vaira ranch (CA, USA)**	C3 Grassland	38.41	-120.95	888	0.3	1
**Kendall (AZ, USA)**	C3/C4 Grassland	31.74	-109.94	389	0.9	4
**Hainich (DE)**	Deciduous Forest	51.08	10.45	1500	1	1.2
**Little Prospect Hill - LPH (MA, USA)**	Mixed Forest	42.54	-72.54	1284	1	2
**Jornada Basin (NM, USA)**	C3/C4 Grassland and Shrubs	32.51	-106.78	218	0.9	4
**TasFACE (AUS)**	C3/C4 Grassland	-42.70	147.26	812	0.2	5

Supplementary Table 2. Simulated long-term average net radiation (R_n), latent heat (λE), sensible heat (H), Gross Primary Production (GPP), Leaf Area Index (LAI) and transpiration (T) with the T&C model in the location of Morgan Monroe Forest for a number of scenarios. The base case (van Genuchten parameterization) without (VG), with soil structure effects (VG+SS) and with soil structure effects and changes in θ_{mac} (VG+SS+ θ_{mac}) represent the reference scenarios. Additional scenarios where the macropore saturated water content (θ_{mac}) is decreased and increased from the base case of -50%, + 100% and + 200% (*0.5, *2 and *4) and the ratio $\alpha_{str}/\alpha_{tex}$ is modified from the base case assuming $\alpha_{str}/\alpha_{tex} = 10$, $\alpha_{str}/\alpha_{tex} = 50$, and $\alpha_{str}/\alpha_{tex} = 100$ are also presented.

	VG	VG+SS	VG+SS+ θ_{mac}	VG+SS+ $0.5*\theta_{mac}$	VG+SS+ $2*\theta_{mac}$	VG+SS+ $4*\theta_{mac}$	VG+SS+ $\alpha_{str}/\alpha_{tex}$=10	VG+SS+ $\alpha_{str}/\alpha_{tex}$=50	VG+SS+ $\alpha_{str}/\alpha_{tex}$=100
R_n [W m^{-2}]	94,6	94,8	94,8	94,8	94,9	95,0	94,8	94,8	94,8
λE [W m^{-2}]	53,4	55,7	55,6	55,7	55,6	55,5	55,4	55,7	55,6
H [W m^{-2}]	38,9	36,6	36,7	36,6	36,8	37,2	36,8	36,6	36,6
GPP [gC m^{-2} yr^{-1}]	1582,0	1582,4	1582,4	1582,4	1582,6	1582,8	1582,4	1581,5	1581,5
LAI [-]	2,1	2,1	2,1	2,1	2,1	2,1	2,1	2,1	2,1
T [mm yr^{-1}]	494,7	489,2	490,3	489,8	491,5	493,9	489,6	488,9	489,1

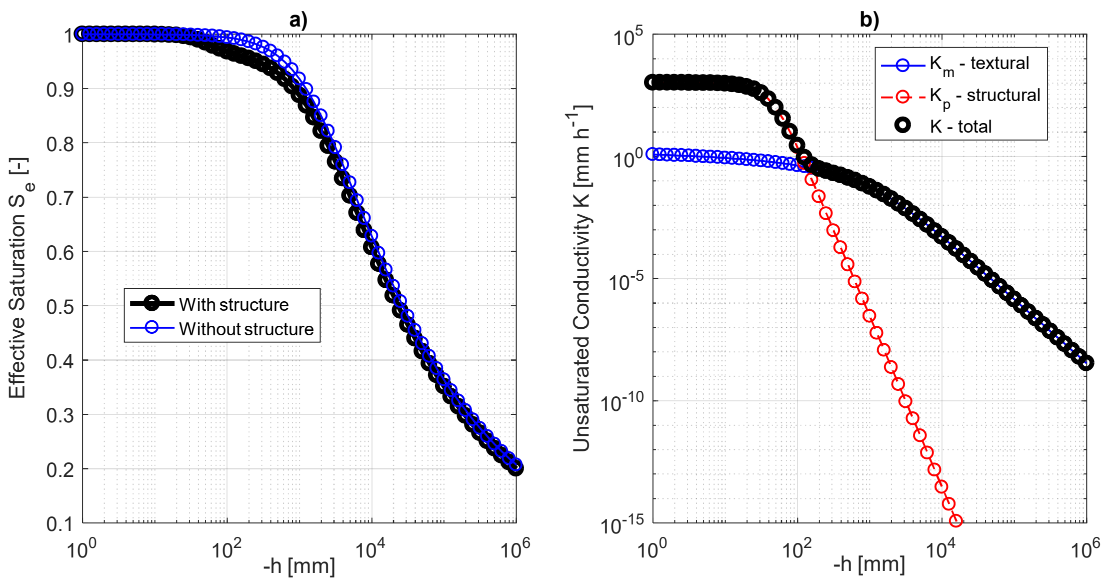

Supplementary Figure 1. a) The soil water retention curve (expressed as effective saturation S_e – water potential h) for the same soil, with and without consideration of structural effects; b) the hydraulic conductivity function of the matric soil (textural component) K_m [mm h^{-1}] and the hydraulic conductivity due to the presence of soil structural features K_p. The total hydraulic conductivity K is the sum of the two components. Soil hydraulic properties correspond to the soil of the Konza Prairie case study.

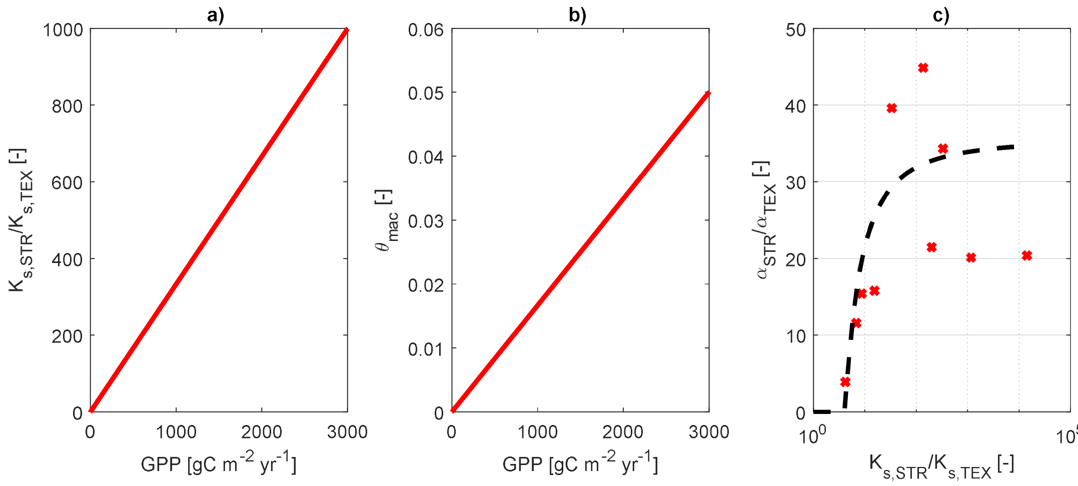

Supplementary Figure 2. Soil structural features computed as a function of Gross Primary Production (GPP) used as proxy of the degree of bioturbation activity. a) The ratio between structural and textural saturated conductivity $K_{s,str}/K_{s,tex}$; b) saturated water content (additional porosity) associated with the presence of structural features θ_{mac}; and c) the relation between $\alpha_{str}/\alpha_{tex}$ as a function of $K_{s,str}/K_{s,tex}$ ($\alpha_{str}/\alpha_{tex} = -14.11*[\log_{10}(K_{s,str}/K_{s,tex})]^{-1.917} + 35.59$, with a minimum threshold of 1 for the $\alpha_{str}/\alpha_{tex}$ value) that has been fitted ($R^2 = 0.38$) using empirical observations (red stars) from Smettem and Kirkby (1990); Kutílek and Jendele (2008); and Coppola et al. (2009a,b).

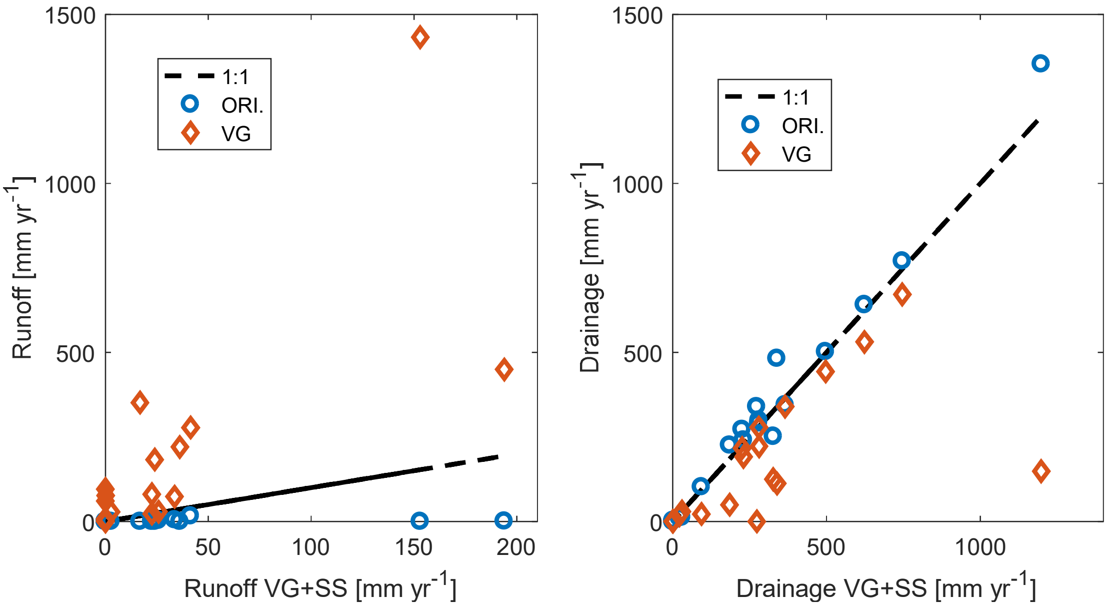

Supplementary Figure 3. Simulated (T&C) long-term ecosystem-scale runoff (a) and water drainage at the bottom of the soil profile (b) for the twenty locations in two scenarios: "VG" soil hydraulic parameter (van Genuchten parameterization) derived from the global soil map (SoilGrids-250m) and without soil structural effects and "ORI" original soil hydraulic parameterization based on local soil textural properties and model tuning. Deviation from the third scenario "VG+SS" are shown. The 1:1 line represents the scenario "VG+SS."

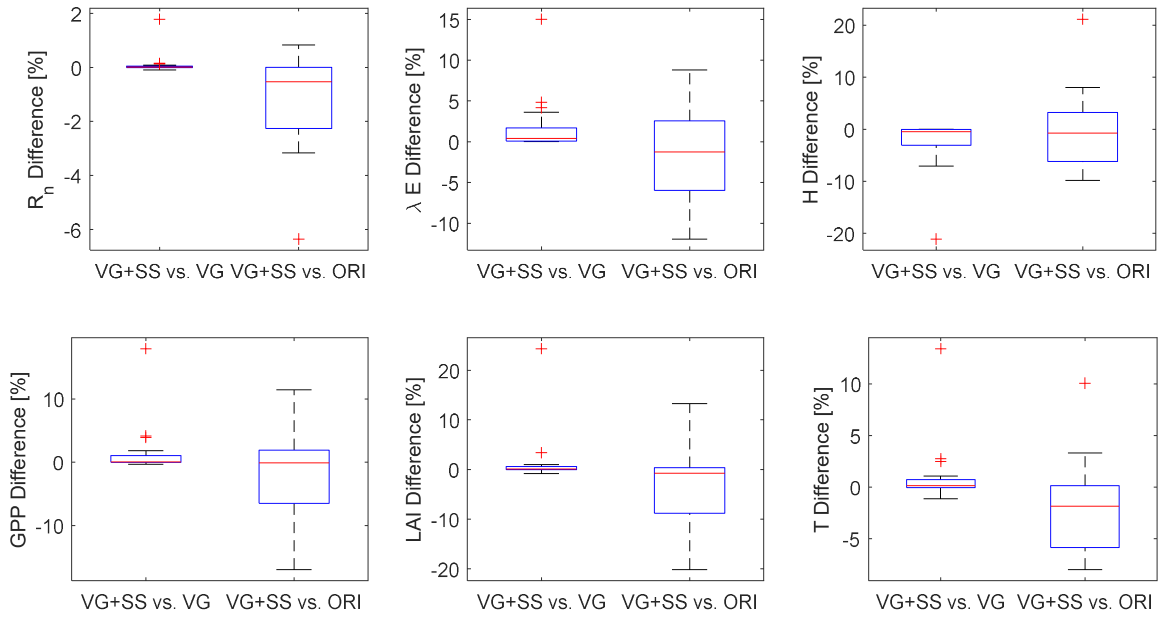

Supplementary Figure 4. Boxplot representation of the percent differences in long-term average net radiation (R_n), latent heat (λE), sensible heat (H), Gross Primary Production (GPP), Leaf Area Index (LAI) and transpiration (T). Boxplots include results for the 20 sites simulated with the T&C model at ecosystem-scale. Differences are shown between the scenario with soil structure parameterization "VG+SS" and the two other scenarios: "VG" and "ORI."

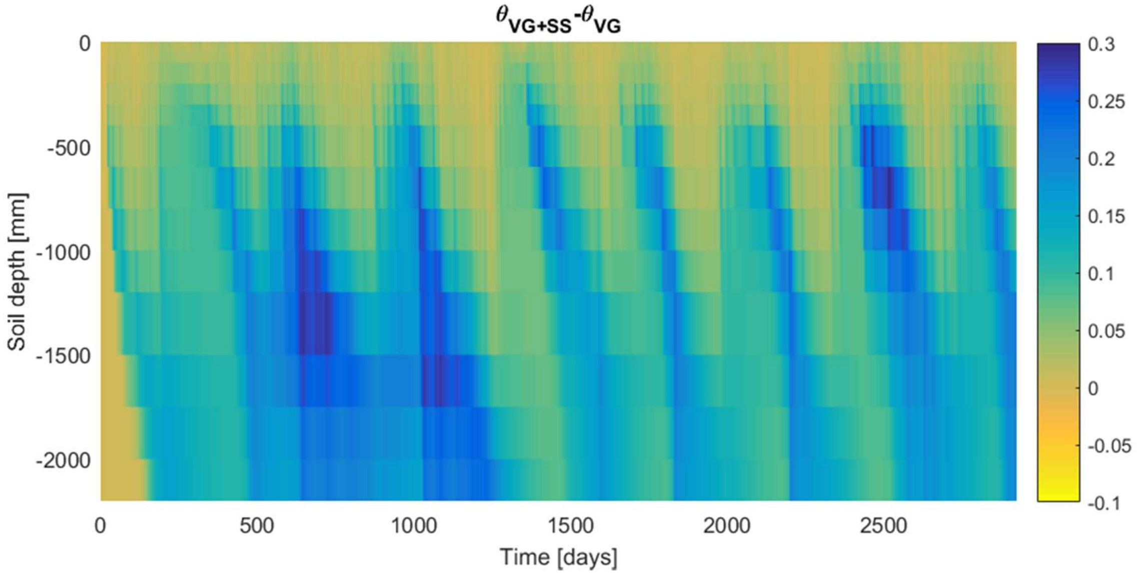

Supplementary Figure 5. Differences in water content (θ) at various depths (z) between the scenario "VG+SS" with soil hydraulic parameter (van Genuchten parameterization) derived from the global soil map (SoilGrids-250m) with soil structural effects and the scenario "VG" soil hydraulic parameter derived from the global map but without soil structural effects. Results correspond to the simulations with T&C at the Morgan Monroe Forest location.

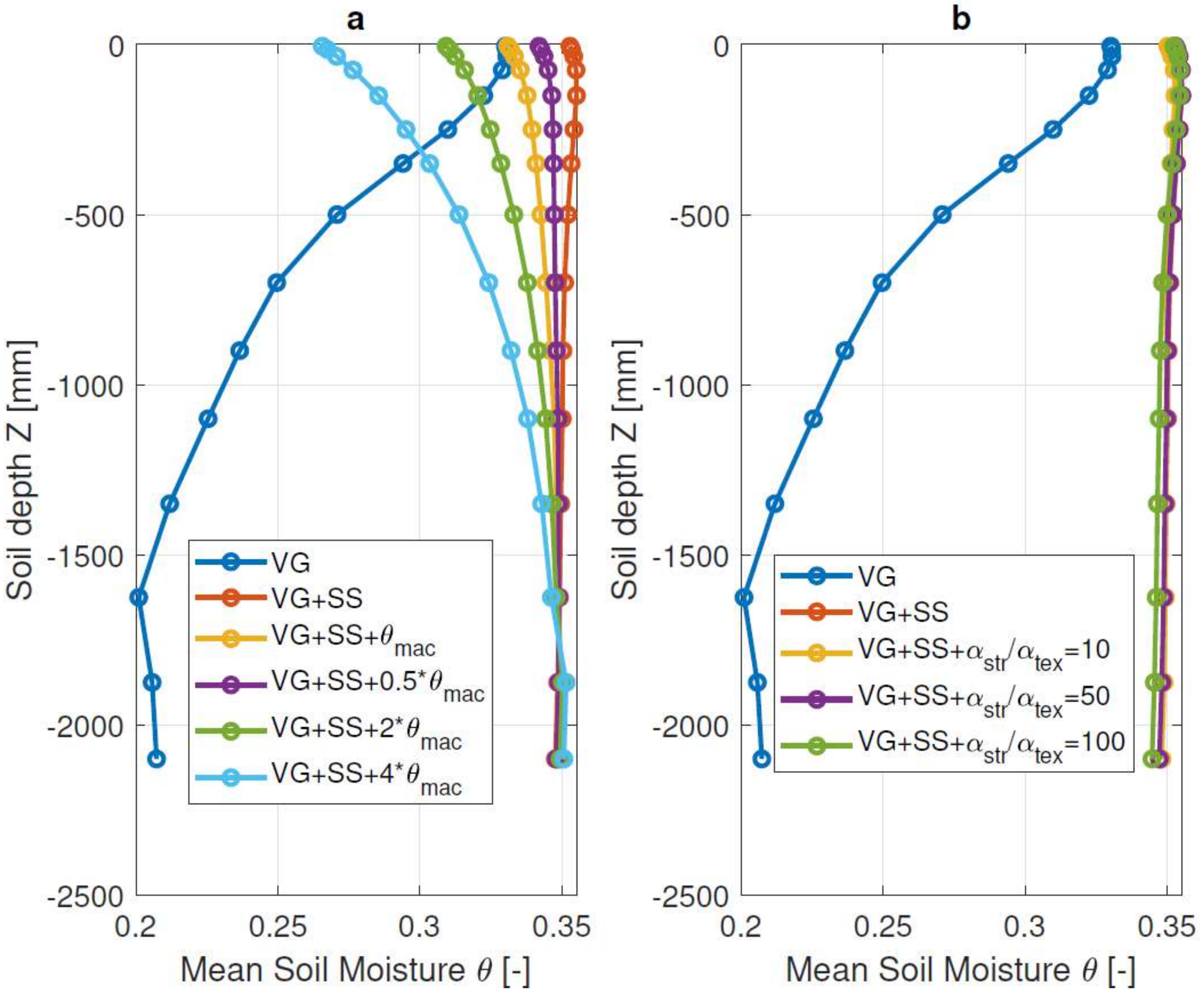

Supplementary Figure 6. Long-term averaged soil moisture θ, profile with soil depth for a number of scenarios. The base case (van Genuchten parameterization) without (VG) and with soil structure effects (VG+SS) are presented in both subplot (a) and (b). In subplot (a) additional scenarios are presented, where a macropore saturated water content θ_{mac} larger than zero is considered and then decreased and increased from the reference value of -50%, + 100% and + 200% (*0.5, *2 and *4). In subplot (b) additional scenarios are presented, where soil structure effects are included and the ratio $\alpha_{str}/\alpha_{tex}$ is modified from the reference value ($\alpha_{str}/\alpha_{tex} = 33$) assuming $\alpha_{str}/\alpha_{tex} = 10$, $\alpha_{str}/\alpha_{tex} = 50$, and $\alpha_{str}/\alpha_{tex} = 100$. Results correspond to simulations with T&C at the location of Morgan Monroe Deciduous Forest.

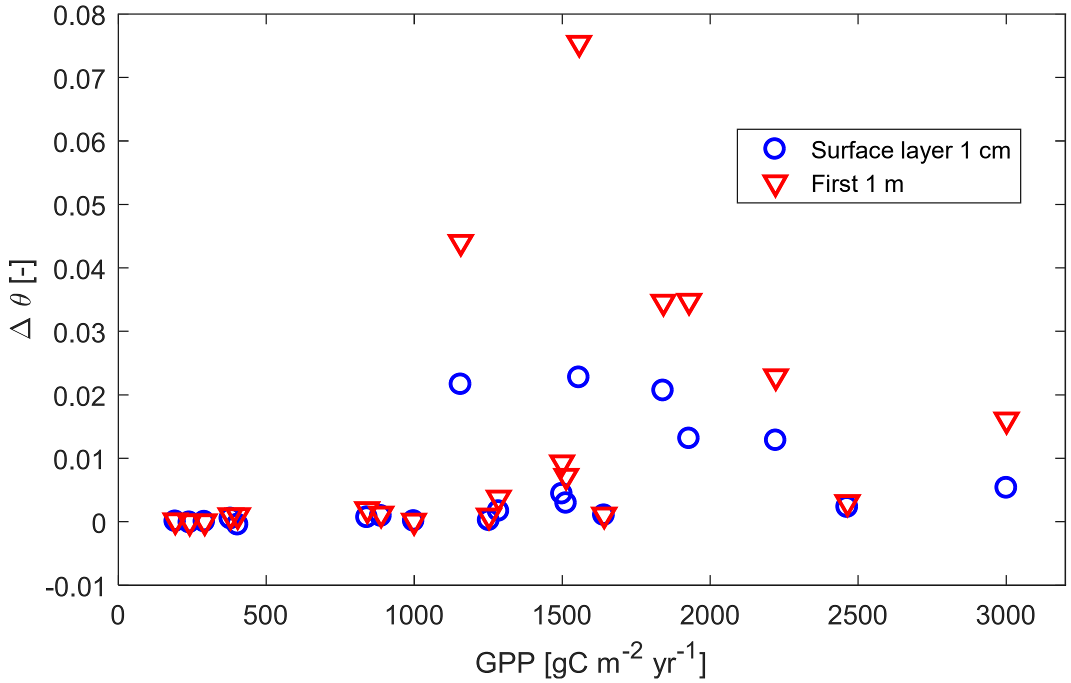

Supplementary Figure 7. Differences in long-term average water content (θ) integrated in the top soil layer (1 cm) and over the first meter of soil (or less if the soil is shallower) between the scenario "VG+SS" with soil hydraulic parameter (van Genuchten parameterization) derived from the global soil map (SoilGrids-250m) with soil structural effects and the scenario "VG" - soil hydraulic parameter derived from the global map but without soil structural effects. The 20 locations are ranked according to their GPP.

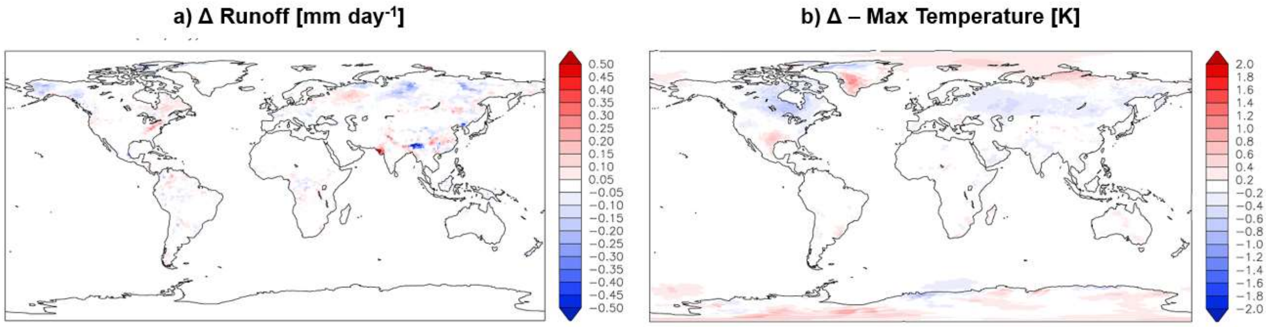

Supplementary Figure 8. Differences in (a) runoff [mm day^{-1}], and (b) daily maximum near-surface temperature [K] averaged over 30 years of simulations, with soil structure (WSS) compared to no soil-structure (NSS), i.e., Δ=WSS-NSS, using the global climate model OLAM.

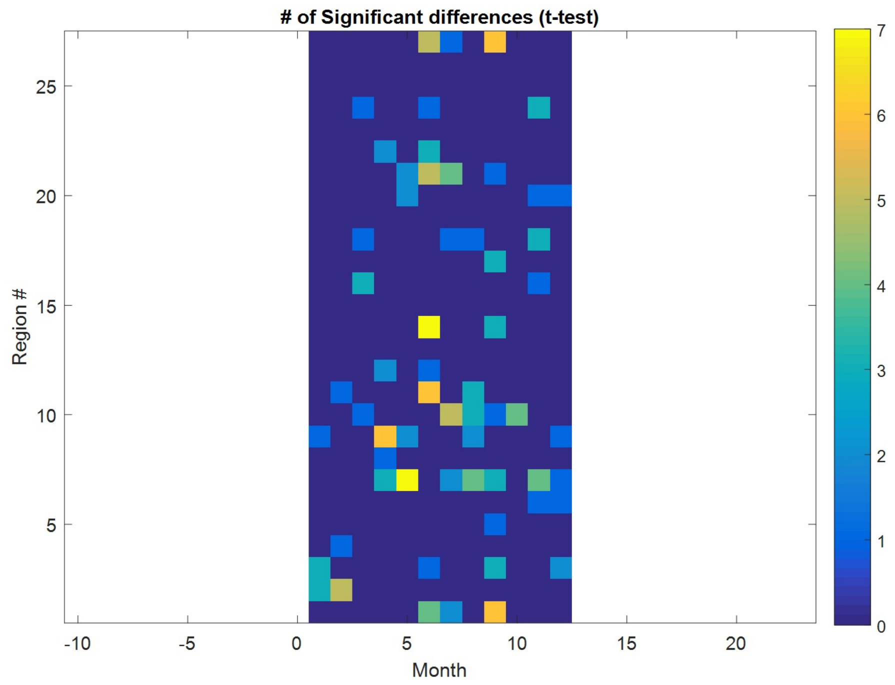

Supplementary Figure 9. For each of 27 regions (Supplementary Figure 12) and month of the year, the number of statistically significant differences (t-test, α =0.05 level of significance) in the sample mean between soil structure (WSS) and no soil structure (NSS) is reported. Maximum number of variables is 11, which comprises the main land-surface and climatic variables (surface short-wave radiation, precipitation, near-surface air temperature, canopy temperature daily range, soil temperature daily range, canopy specific humidity, surface net radiation, surface sensible heat flux, surface latent heat flux, skin surface temperature, and canopy temperature).

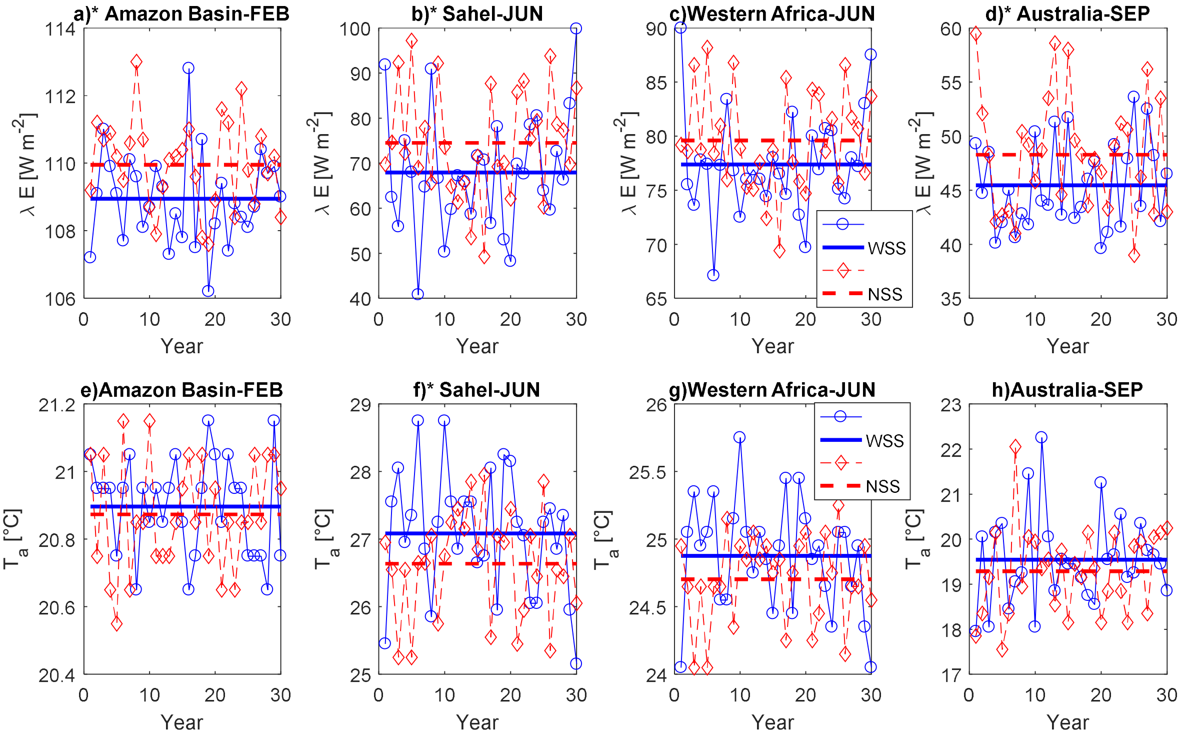

Supplementary Figure 10. Average surface latent heat flux [W m^{-2}] and canopy air temperature [°C] for selected regions and months. The average of the month for each year is shown over the Amazon basin in February (a, e), Sahel in June (b, f), Western Africa in June (c, g) and Australia in September (d, h). Statistically significant differences in the long-term mean are marked with an asterisk (*). Results are reported for the scenario that does not account for any soil structural effects (NSS) and for the scenario with soil structural effects (WSS). Horizontal lines represent the long-term monthly mean for the WSS and NSS scenarios.

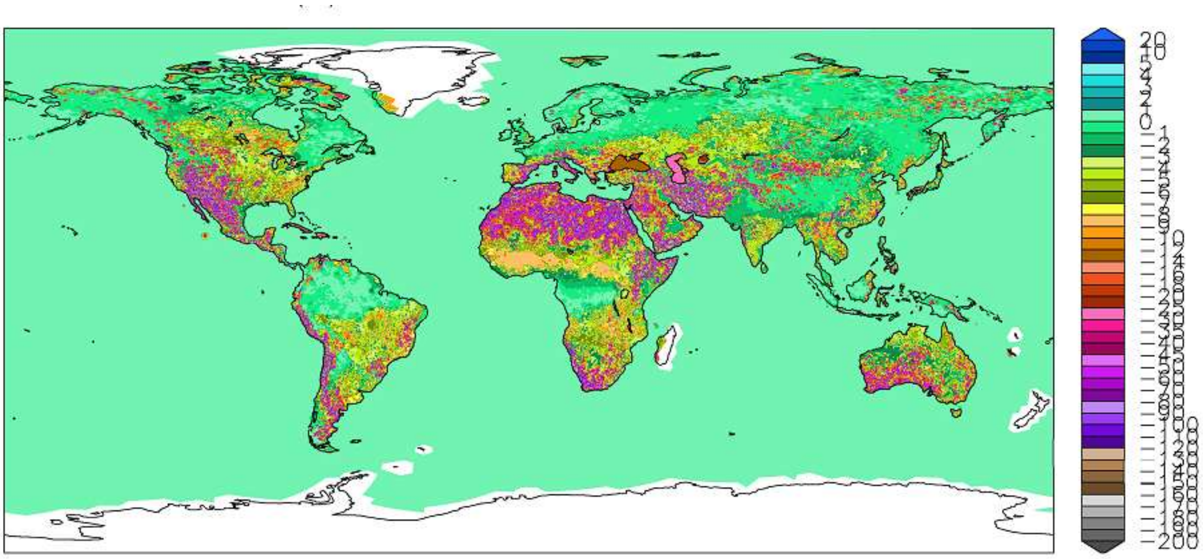

Supplementary Figure 11. Water table depth [m] after 35 years of simulation with soil structure.

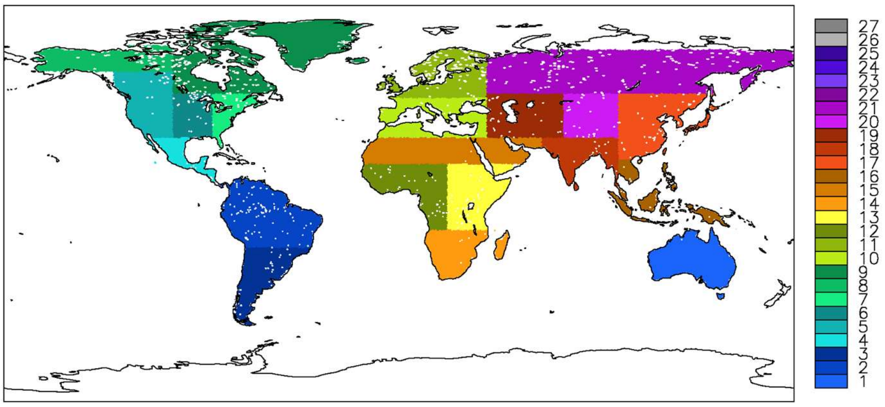

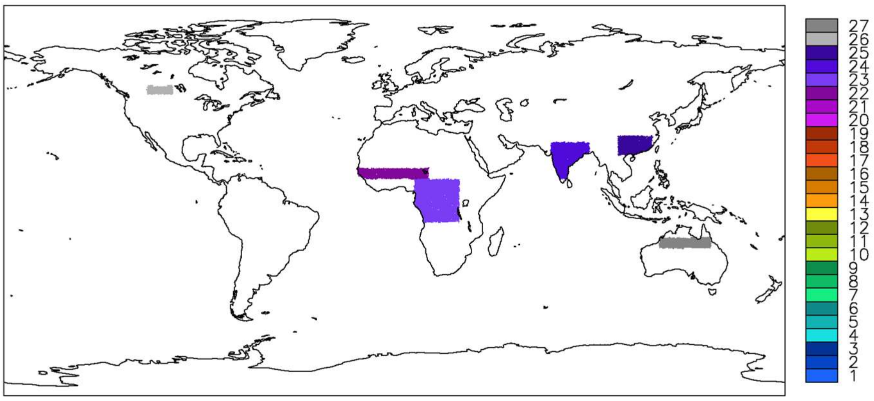

Supplementary Figure 12. Subdivision of the globe for the analysis in 27 geographical regions. Regions from 22 to 27 are nested in some of the previous 21 regions. The 27 geographical regions are: 1 Australia [Latitude 45S, 11S; Longitude 110E, 155E], 2 Amazon Basin [Latitude 20S, 12N; Longitude 82W, 34W], 3 Southern South America [Latitude 56S, 20S; Longitude 76W, 40W], 4 Central America [Latitude 10N, 30N; Longitude 116W, 83W], 5 Western North America [Latitude 30N, 60N; Longitude 130W, 103W], 6 Central North America [Latitude 30N, 50N; Longitude 103W,85W], 7 Eastern North America [Latitude 25N, 50N; Longitude 85W, 60W], 8 Alaska [Latitude 60N, 72N; Longitude 170W, 103W], 9 Greenland [Latitude 50N, 85N; Longitude 103W, 10W], 10 Mediterranean Basin [Latitude 30N, 48N; Longitude 10W,40E], 11 Northern Europe [Latitude 48N, 75N; Longitude 10W,40E], 12 Western Africa [Latitude 12S, 18N; Longitude 20W, 22E], 13 Eastern Africa [Latitude 12S, 18N; Longitude 22E, 52E],14 Southern Africa [Latitude 35S, 12S; Longitude 10W, 52E, 15 Sahara [Latitude 18N, 30N; Longitude 20W, 65E],16 Southeast Asia [Latitude 11S, 20N; Longitude 95E, 155E, 17 East Asia [Latitude 20N, 50N; Longitude 100E, 145E],18 South Asia [Latitude 5N, 30N; Longitude 65E,100E], 19 Central Asia [Latitude 30N, 50N; Longitude 40E, 75E], 20 Tibet [Latitude 30N, 50N; Longitude 75E, 100E], 21 North Asia [Latitude 50N, 70N; Longitude 40E, 180E], 22 Sahel [Latitude 10N, 15N; Longitude 40W, 16E], 23 Central Africa [Latitude 10S, 10N; Longitude 9E, 30E], 24 India [Latitude 10N, 27N; Longitude 72E, 90E],

25 South China [Latitude 21N, 30N; Longitude 103E,119E], 26 Canadian Plains [Latitude 49N, 53N; Longitude 114W,102W], 27 North Australia [Latitude 22S, 17S; Longitude 122E, 146E].
